# Associations in Both Directions Between Internet Gaming Disorder and Suicidal Ideation and Attempts Among Chinese Adolescent Gamers: Two-Wave Prospective Cohort Study

**DOI:** 10.2196/86919

**Published:** 2026-09-02

**Authors:** Pu Peng, Zhangming Chen, Silan Ren, Yudiao Liang, Youguo Tan, Xiaogang Chen, Jinsong Tang, Yanhui Liao

**Affiliations:** 1 Zhejiang University School of Medicine Department of Psychiatry, Sir Run Run Shaw Hospital Hangzhou China; 2 Department of Psychiatry, Zigong Mental Health Center Zigong China; 3 Department of Nursing, Sichuan Vocational College of Health and Rehabilitation Sichuan China; 4 Second Xiangya Hospital of Central South University Changsha China

**Keywords:** internet gaming disorder, suicidal ideation, suicidal attempt, adolescent, longitudinal

## Abstract

**Background:**

Internet gaming disorder (IGD) has been associated with suicidal outcomes in adolescents, but most evidence is cross-sectional or has examined only whether IGD predicts later suicidality. Whether suicidal ideation and suicidal attempts are also associated with subsequent IGD remains unclear.

**Objective:**

This study aimed to examine prospective associations in both directions between IGD and suicidal ideation and suicidal attempts among Chinese adolescent gamers.

**Methods:**

This school-based, 2-wave prospective cohort study was conducted in Zigong, China, with assessments in November 2021 and November 2022. The analytic sample included 57,985 adolescents who completed both waves and reported gaming in the past 12 months at baseline. Students were recruited through school-based cluster sampling and completed electronic questionnaires during school sessions. IGD was assessed using the 9-item Internet Gaming Disorder Scale-Short Form (IGDS9-SF), with probable IGD defined as a score of 32 or higher. Suicidal ideation referred to serious consideration of suicide during the past month, whereas suicidal attempt was assessed over the lifetime. Both outcomes were assessed at both waves. Incident outcomes were defined among participants without the corresponding outcome at baseline. Logistic regression models estimated cross-sectional and prospective associations with sequential adjustment for demographic, lifestyle, and mental health covariates.

**Results:**

At baseline, 1333 of 57,985 adolescents had IGD (2.3%, 95% CI 2.2%-2.4%), 8450 had suicidal ideation (14.6%, 95% CI 14.3%-14.9%), and 6666 reported a suicidal attempt (11.5%, 95% CI 11.2%-11.8%). IGD was associated with higher baseline prevalence of suicidal ideation (crude odds ratio [COR] 6.45, 95% CI 5.78-7.20, *P*<.001; adjusted odds ratio [AOR] 1.50, 95% CI 1.31-1.72, *P*<.001) and suicidal attempt (COR 5.38, 95% CI 4.81-6.02, *P*<.001; AOR 1.48, 95% CI 1.29-1.69, *P*<.001). Prospectively, baseline IGD was associated with incident suicidal ideation (COR 2.49, 95% CI 2.02-3.06, *P*<.001; AOR 1.38, 95% CI 1.11-1.73, *P*=.004) and incident suicidal attempt (COR 2.99, 95% CI 2.47-3.61, *P*<.001; AOR 1.37, 95% CI 1.12-1.68, *P*=.002). Conversely, baseline suicidal ideation was associated with incident IGD (COR 2.98, 95% CI 2.58-3.45, *P*<.001; AOR 1.37, 95% CI 1.14-1.64, *P*<.001), as was baseline suicidal attempt (COR 2.63, 95% CI 2.24-3.08, *P*<.001; AOR 1.37, 95% CI 1.14-1.64, *P*<.001).

**Conclusions:**

This study is innovative in testing prospective associations in both directions within an adolescent gamer cohort while examining suicidal ideation and suicidal attempts separately. Unlike previous studies that were predominantly cross-sectional or treated IGD only as a predictor of later suicidality, it identifies suicidal outcomes as prospective predictors of incident IGD. The findings add longitudinal evidence that IGD and suicidality are interrelated prevention targets, although they do not establish causality. In practice, school-based and clinical screening should assess problematic gaming, suicide risk, and co-occurring mental health symptoms together, with timely referral and integrated intervention for adolescents who screen positive in either domain.

## Introduction

Adolescent suicide constitutes a severe global public health challenge and ranks as the second leading cause of mortality for individuals aged 10-24 years globally [1]. This crisis is particularly acute in China, where recent epidemiological data reveal a disturbing divergence in suicide trends. While rates have declined in most age groups, they have steadily increased since 2011 among children aged 5-14 years and have risen sharply since 2017 among adolescents aged 15-24 years [2]. This alarming pattern underscores an urgent imperative to identify and understand the novel and modifiable risk factors driving this specific demographic shift.

In parallel, the proliferation of digital media has spurred clinical and research concern regarding internet gaming disorder (IGD) and its potential role in adolescent psychopathology [3-8]. A growing body of primarily cross-sectional evidence consistently links IGD to suicidal behaviors [9-11]. Systematic reviews encompassing diverse cultural contexts have established a reliable association between IGD and heightened suicidal ideation [12], with further studies corroborating a link to suicidal attempts [13-15]. Clinical case reports provide a mechanistic glimpse, suggesting that crises leading to suicidality in adolescents with IGD are often precipitated by conflicts over gaming or attempts to restrict access [16,17].

Theoretical models posit that the IGD-suicidality association might be bidirectional. The integrated motivational-volitional model of suicidal behavior provides a useful framework for conceptualizing how IGD may be linked to suicidal ideation and suicidal attempt [18]. It distinguishes three phases: a premotivational phase involving environmental and personal risk factors, a motivational phase in which feelings such as defeat and entrapment give rise to suicidal ideation, and a volitional phase that determines whether individuals act on these thoughts. In the premotivational phase, excessive gaming and its consequences, such as stressful life events, academic impairment, family conflict, social withdrawal, and sleep disturbance, may increase chronic stress and defeat experiences [19,20]. In the motivational phase, adolescents with IGD may use gaming as an escape-oriented coping strategy for negative affect; although this may provide short-term relief, it may also delay problem solving, weaken resilience and emotional regulation, and deepen feelings of entrapment [21-23], thereby increasing vulnerability to suicidal ideation. In the volitional phase, the transition from suicidal ideation to suicidal attempt may be facilitated by behavioral dysregulation, impulsivity, and impaired executive control, which are commonly linked to IGD [24-26]. In addition, prolonged exposure to video games, especially violent games, may desensitize players to pain, thereby increasing pain tolerance and the “acquired capability” for suicide necessary for suicidal attempts [27-29].

In the opposing direction, theoretical frameworks like the compensatory internet use model and the interaction of person-affect-cognition-execution (I-PACE) model support a reverse pathway, positing that suicidality may precipitate IGD [30,31]. These frameworks suggest that preexisting psychological distress, including suicidal ideation, may drive adolescents to engage in gaming as a maladaptive coping mechanism to escape aversive emotional states, thereby increasing vulnerability to disordered use. This proposed bidirectionality is empirically precedented; longitudinal studies have indicated a reciprocal relationship between IGD and depression [32,33], suggesting that a similar dynamic may exist for the more severe outcome of suicidal behavior.

Despite this compelling theoretical rationale, a critical empirical gap persists. The vast majority of extant literature is limited by its cross-sectional design, which is inherently incapable of establishing temporal precedence or causality. While a handful of longitudinal studies have begun to emerge, these studies have predominantly examined a one-way relationship, positioning IGD only as a predictor of future suicidality, thereby overlooking the potential reverse association [34-38]. This narrow focus overlooks the clinically crucial possibility that suicidality may itself be a potent risk factor for the development of IGD. A hint of this complex interplay was observed in our recent longitudinal network analysis of depressive symptoms and IGD, which identified suicidal ideation as a central bridge symptom within the IGD-depression network [33]. However, this finding was incidental, as the study was not designed to formally test the hypothesis that baseline suicidal behaviors prospectively predict the new onset of IGD.

To address this research gap, we conducted a large-scale, school-based, 2-wave prospective cohort study among Chinese adolescents. We aim to move beyond correlation and unidirectional assumptions to explicitly test for a bidirectional relationship. This study has two primary objectives: (1) to determine if baseline IGD predicts the new onset of suicidal behaviors after 1 year, adjusting for critical mental health confounders (ie, depression, anxiety, sleep disturbance, and externalizing problems); and (2) to test the reverse hypothesis, examining if baseline suicidal behaviors predict subsequent incident IGD. By elucidating the temporal dynamics of this relationship, our findings have the potential to inform more nuanced and effective early intervention and prevention strategies for both suicidal behavior and IGD.

## Methods

### Study Design and Setting

This was a school-based, 2-wave prospective cohort study conducted in Zigong City, China. The baseline assessment was conducted in November 2021, and the follow-up assessment was conducted in November 2022. Data were collected in public middle schools using standardized electronic questionnaires completed during regular school sessions. Part of the study data has been published elsewhere [10,33].

### Sampling Procedures and Participants

A 2-stage cluster sampling procedure was used. First, all public middle schools in Zigong City were invited to participate. Second, all students from participating schools were invited to complete the survey. The questionnaire was administered in school computer laboratories during a 45-minute class session under the supervision of teachers who had received standardized training on the survey procedure.

### Inclusion and Exclusion Criteria

Participants were eligible for the present analyses if they had valid baseline and follow-up responses, successful linkage across the 2 waves, and past-year gaming behavior at baseline. Responses were excluded during quality control if they had invalid identifiers, implausible completion times, or logical inconsistencies, such as implausible age values. Because the analytic sample was restricted to adolescents with baseline past-year gaming behavior, the findings apply to adolescent gamers rather than to the general adolescent population.

### Participant Characteristics

**Figure 1:**
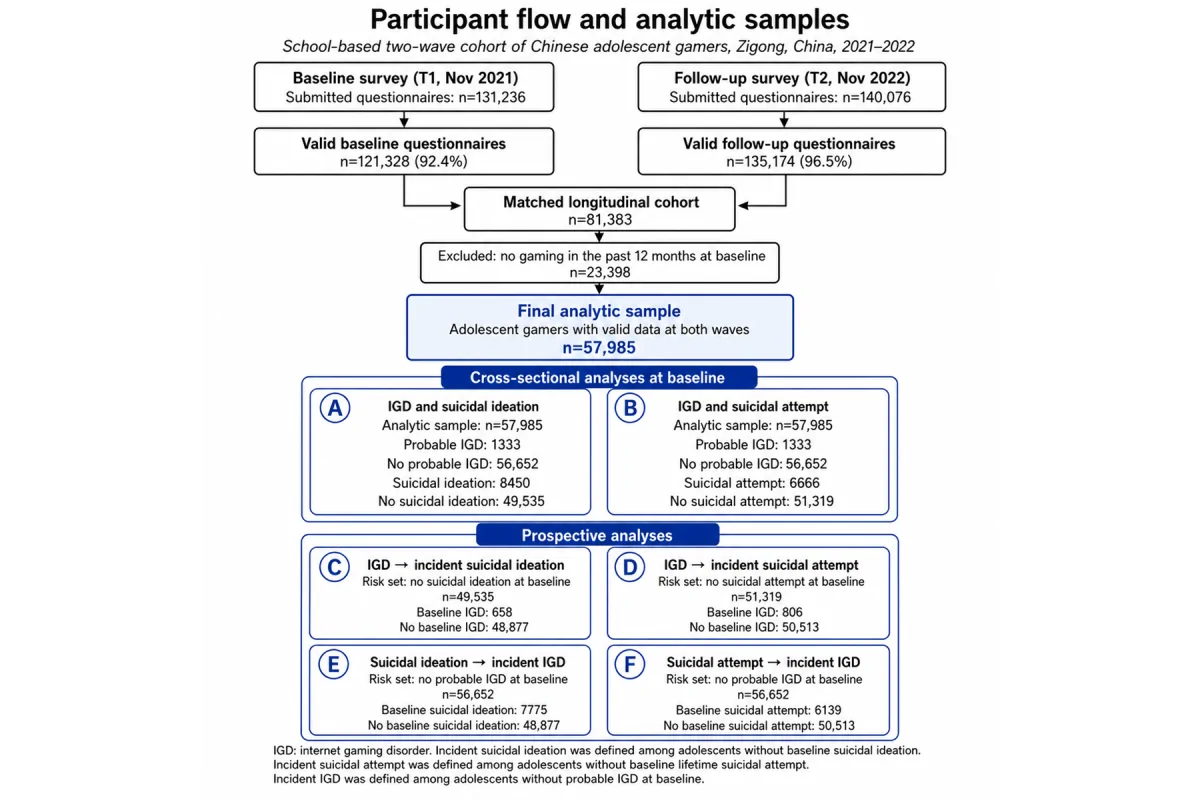
Study flowchart of a school-based two-wave cohort of Chinese adolescent gamers in Zigong, China (2021–2022).

As shown in Figure 1, from initial pools of 131,236 (T1) and 140,076 (T2) submissions, valid responses were obtained from 121,328 (92.4%) and 135,174 (96.5%) students, respectively. Matching student IDs yielded a longitudinal cohort of 81,383 participants. Attrition analysis comparing the longitudinal sample with those lost to follow-up revealed statistically significant but negligible differences in baseline IGD (12.7%, 10,304/81,383 in the follow-up group versus 14.8%, 5898/39,945 in the lost-follow-up group, Cramer’s V=0.017) and suicidal behaviors (17.7%, 14,415/81,383 in the follow-up group versus 19.2%, 7655/39,945 in the lost-follow-up group, Cramer’s V=0.017). The detailed comparison could be found in Table S1 in Multimedia Appendix 1.

### Data Collection and Data Quality

All data were collected using self-administered electronic questionnaires. Participants could withdraw from the survey at any time; however, the electronic questionnaire could only be submitted after all required items had been completed. Therefore, among valid submitted questionnaires, there were no item-level missing data for the exposure, outcomes, or covariates used in the present analyses. Data quality checks were conducted before analysis, including checks for valid identifiers, reasonable completion time, and logical consistency.

### Measurements and Covariates

#### IGD

IGD symptoms were assessed using the 9-item Internet Gaming Disorder Scale-Short Form (IGDS9-SF), with total scores ranging from 9 to 45 [39,40]. In the primary analyses, probable IGD was defined as an IGDS9-SF score of 32 or higher, based on a validation study in Chinese adolescents that reported high diagnostic accuracy for this threshold (sensitivity, 98%; specificity, 91.9%) [41]. A lower cutoff of 21, which has been commonly used in previous epidemiological studies to identify problematic gaming symptoms [42,43], was used in sensitivity analyses. The scale demonstrated strong internal consistency in our sample at both baseline (α=0.899) and follow-up (α=0.840).

#### Suicidal Ideation and Attempt

Suicidal outcomes were assessed at both baseline and follow-up using 2 self-report items. Suicidal ideation was assessed by asking whether participants had seriously considered suicide during the past month. Suicidal attempt was assessed by asking whether participants had ever attempted suicide in their lifetime. Suicidal ideation and suicidal attempt were analyzed separately because they differ in time frame, severity, and clinical interpretation. This methodological approach is consistent with established practices in large-scale epidemiological surveys of suicidality [44-46]. In prospective analyses, incident suicidal ideation was defined as no suicidal ideation at baseline and suicidal ideation at follow-up. Incident suicidal attempt was operationalized among adolescents who reported no lifetime suicidal attempt at baseline; a positive lifetime suicidal attempt response at follow-up was coded as an incident suicidal attempt during follow-up. The exact timing of the attempt within the 1-year follow-up interval was not assessed.

#### Covariates

Covariates comprised key demographic and lifestyle factors: age, sex, educational stage (junior high, senior high, vocational), residence (urban or rural), family structure (nuclear vs single-parent or remarried), and status as an only child or left-behind child, alongside parental education level and recent smoking or drinking behaviors. Mental health problems were assessed using validated questionnaires: the 9-item Patient Health Questionnaire (PHQ-9) for depression [47], the 7-item Generalized Anxiety Disorder Questionnaire (GAD-7) for anxiety [48], the Pittsburgh Sleep Quality Index (PSQI) for sleep disturbance [49], and the conduct problem and hyperactivity and inattention subscales derived from the Strengths and Difficulties Questionnaire (SDQ). A cutoff score of 10 for PHQ-9, 10 for GAD-7, 6 for PSQI, 5 for the SDQ conduct problem subscale, and 7 for the SDQ hyperactivity and inattention subscale was used to determine the presence of mental health problems [49-52].

### Sample Size, Power, and Precision

No formal a priori power calculation was conducted because this study used data from a large population-based school cohort. The achieved analytic sample size was determined by the number of participants with valid baseline data, valid follow-up data, successful linkage across waves, and baseline past-year gaming behavior. Precision was evaluated using 95% CIs for the main association estimates.

### Statistical Analysis

Baseline and follow-up data from 57,985 adolescent gamers who completed both assessments were analyzed. All statistical analyses were performed using R version 4.2.0 (R Foundation for Statistical Computing), and 2-sided *P* values were reported. Baseline characteristics were compared between adolescents with and without IGD. Continuous variables were compared using t tests, and categorical variables were compared using chi-square tests. The Bonferroni-corrected threshold for baseline comparisons was *P*<.003, based on 18 comparisons between adolescents with and without IGD.

For cross-sectional and prospective association analyses, logistic regression models were fitted using sequential adjustment. Model 0 was unadjusted. Model 1 adjusted for demographic variables, including age, sex, age group, only-child status, left-behind status, residence, family type, and parental education. Model 2 additionally adjusted for lifestyle variables, including alcohol use and tobacco or nicotine use. Model 3 additionally adjusted for mental health variables, including anxiety, sleep disturbance, depression, hyperactivity and inattention, and conduct problems. Absolute prevalence or risk differences were reported in percentage points to aid interpretation of practical significance and were calculated as the outcome prevalence or incidence in the exposed group minus that in the unexposed group. Although the original survey used a school-based cluster sampling procedure, school identifiers were removed during data cleaning and de-identification before construction of the analytic dataset for the present study. Therefore, clustering at the school could not be modeled. The regression models should therefore be interpreted as individual-level analyses.

Subgroup analyses were conducted by sex, only-child status, left-behind status, residence, family type, and age group. Interaction terms between the exposure and each subgroup variable were tested to assess potential effect modification. These analyses were considered exploratory. Sensitivity analyses were performed by using an alternative cutoff point of 21 on the IGDS9-SF to capture broader problematic gaming.

### Missing Data

The electronic questionnaire required completion of all required items before submission. Therefore, among valid submitted questionnaires, there were no item-level missing data for the exposure, outcomes, or covariates included in the present analyses. All regression analyses were conducted using complete cases. Potential attrition-related bias was assessed by comparing baseline characteristics between participants included in and lost from the longitudinal sample. Because no item-level missingness was present in the analytic dataset, missing completely at random (MCAR) testing and multiple imputation were not performed.

### Ethical Considerations

This research was conducted in accordance with the Declaration of Helsinki and received ethical approval from the Zigong Mental Health Center Ethics Committee [Number 2021003]. Written informed consent was obtained from all participants, with parental consent required for those under 18. All data used for analysis were de-identified before analysis, and results are reported only in aggregate form. No individually identifiable participant information is presented in the manuscript, tables, figures, or supplementary materials. Participants received no financial compensation for participation. Following the survey, psychological crisis intervention training was provided to psychology teachers to help identify and support high-risk students. Psychological counseling services were made available to all participants, with encouragement for both students and parents to use these resources. Students endorsing recent suicidal ideation or otherwise identified as having severe mental distress received confidential follow-up from trained school psychological counselors or teachers. Those requiring professional assessment were promptly referred to the Zigong Mental Health Center. Guardians of at-risk students were informed confidentially and advised to seek professional mental health care for the student.

## Results

### Sample Characteristics

The analytic sample included 57,985 adolescents who reported gaming in the past 12 months at baseline. The mean age was 14.12 (SD 1.45) years, and 31,643 (54.6%) participants were boys. Using the validated IGDS9-SF cutoff of 32 or higher, 1333 (2.3%) adolescents met the criterion for probable IGD.

Baseline characteristics according to IGD status are shown in Table 1. After Bonferroni correction, adolescents with probable IGD differed from those without probable IGD in age, age group, left-behind status, family type, lifestyle factors, mental health indicators, and suicidal outcomes. No corrected differences were observed for sex, residence, only-child status, or parental education.

**Table 1:** Baseline characteristics of Chinese adolescent gamers by internet gaming disorder status in Zigong, China (2021).

Characteristics					Total (N=57,985)			Non-IGD^a^ (n=56,652)			IGD (n=1333)			*P* value^b^	
Demographic characteristics															
	Age, years, mean (SD)			14.12 (1.45)			14.12 (1.45)			13.93 (1.39)			<.001		
Age group, n (%)															<.001
	Early adolescence			35,182 (60.7)			34,286 (60.5)			896 (67.2)					
	Late adolescence			22,803 (39.3)			22,366 (39.5)			437 (32.8)					
Sex, n (%)															.02^c^
	Male			31,643 (54.6)			30,875 (54.5)			768 (57.6)					
	Female			26,342 (45.4)			25,777 (45.5)			565 (42.4)					
Residence, n (%)															.44
	Rural			35,300 (60.9)			34,502 (60.9)			798 (59.9)					
	Urban			22,685 (39.1)			22,150 (39.1)			535 (40.1)					
Only child, n (%)															.01^c^
	No			43,264 (74.6)			42,310 (74.7)			954 (71.6)					
	Yes			14,721 (25.4)			14,342 (25.3)			379 (28.4)					
Left-behind child, n (%)															<.001
	No			40,922 (70.6)			40,051 (70.7)			871 (65.3)					
	Yes			17,063 (29.4)			16,601 (29.3)			462 (34.7)					
Family type, n (%)															<.001
	Nuclear family			45,063 (77.7)			44,164 (78.0)			899 (67.4)					
	Nonnuclear family			12,922 (22.3)			12,488 (22.0)			434 (32.6)					
Father’s education, n (%)															.47
	Below high school			43,425 (74.9)			42,438 (74.9)			987 (74.0)					
	High school or above			14,560 (25.1)			14,214 (25.1)			346 (26.0)					
Mother’s education, n (%)															.92
	Below high school			45,129 (77.8)			44,090 (77.8)			1,039 (77.9)					
	High school or above			12,856 (22.2)			12,562 (22.2)			294 (22.1)					
Lifestyle factors															
	Alcohol use, n (%)														<.001
		No	44,793 (77.3)			43,971 (77.6)			822 (61.7)						
		Yes	13,192 (22.7)			12,681 (22.4)			511 (38.3)						
	Smoking, n (%)														<.001
		No	52,762 (91)			51,835 (91.5)			927 (69.5)						
		Yes	5223 (9)			4817 (8.5)			406 (30.5)						
Mental health indicators															
	Anxiety, n (%)														<.001
		No	51,820 (89.4)			51,218 (90.4)			602 (45.2)						
		Yes	6165 (10.6)			5,434 (9.6)			731 (54.8)						
	Sleep disturbance, n (%)														<.001
		No	38,974 (67.2)			38,663 (68.3)			311 (23.3)						
		Yes	19,011 (32.8)			17,989 (31.8)			1022 (76.7)						
	Depression, n (%)														<.001
		No	47,214 (81.4)			46,833 (82.7)			381 (28.6)						
		Yes	10,771 (18.6)			9,819 (17.3)			952 (71.4)						
	Attention-deficit/hyperactivity disorder symptoms, n (%)														<.001
		No	51,842 (89.4)			51,191 (90.4)			651 (48.8)						
		Yes	6143 (10.6)			5,461 (9.6)			682 (51.2)						
	Conduct problems, n (%)														<.001
		No	53,320 (92)			52,491 (92.7)			829 (62.2)						
		Yes	4665 (8)			4,161 (7.3)			504 (37.8)						
Suicidal behaviors															
	Suicidal ideation, n (%)														<.001
		No	49,535 (85.4)			48,877 (86.3)			658 (49.4)						
		Yes	8450 (14.6)			7775 (13.7)			675 (50.6)						
	Suicidal attempt, n (%)														<.001
		No	51,319 (88.5)			50,513 (89.2)			806 (60.5)						
		Yes	6666 (11.5)			6139 (10.8)			527 (39.5)						

^a^IGD: internet gaming disorder; probable internet gaming disorder was defined using the cutoff point of 32.

^b^*P* values were derived from *t* tests for continuous variables and chi-square tests for categorical variables. The Bonferroni-corrected threshold for baseline comparisons was *P*<.003.

^c^*P*=.02 for sex and *P*=.01 for only-child status; neither was significant after Bonferroni correction.

Adolescents with IGD were slightly younger than those without (13.93 vs 14.12 years) and were more likely to be in early adolescence (67.2%, 896/1333 vs 60.5%, 34,286/56,652). They were also more likely to be left-behind children (34.7%, 462/1333 vs 29.3%, 16,601/56,652) and to live in nonnuclear families (32.6%, 434/1333 vs 22%, 12,488/56,652). Lifestyle risk behaviors were more common in the IGD group, including alcohol use (38.3%, 511/1333 vs 22.4%, 12,681/56,652) and smoking (30.5%, 406/1333 vs 8.5%, 4817/56,652).

Marked differences were observed for mental health indicators. Compared with adolescents without IGD, those with IGD reported higher rates of anxiety (54.8%, 731/1333 vs 9.6%, 5434/56,652), sleep disturbance (76.7%, 1022/1333 vs 31.8%, 17,989/56,652), depression (71.4%, 952/1333 vs 17.3%, 9819/56,652), hyperactivity and inattention (51.2%, 682/1333 vs 9.6%, 5461/56,652), and conduct problems (37.8%, 504/1333 vs 7.3%, 4161/56,652).

### Cross-Sectional Association of IGD With Suicidal Behavior

Table 2 presents cross-sectional findings at baseline. Suicidal ideation and suicidal attempt were substantially more prevalent among adolescents with IGD than among those without IGD.

**Table 2:** Cross-sectional associations between internet gaming disorder and suicidal ideation and attempt among Chinese adolescent gamers in Zigong, China (2021).

Outcome and group		Outcome, n/N (%)	Absolute prevalence difference^a^, percentage points (95% CI)	Model	OR^b^ or AOR^c^ (95% CI)
Suicidal ideation					
	IGD^d^	675/1333 (50.6)	36.9 (34.2-39.6)	Model 0^e^	6.45 (5.78-7.20)^f^
	No IGD	7775/56,652 (13.7)	—^g^	Model 1^h^	6.90 (6.15-7.73)^f^
	—	—	—	Model 2^i^	5.35 (4.74-6.03)^f^
	—	—	—	Model 3^j^	1.50 (1.31-1.72)^f^
Suicidal attempt					
	IGD	527/1333 (39.5)	28.7 (26.1-31.3)	Model 0	5.38 (4.81-6.02)^f^
	No IGD	6139/56,652 (10.8)	—	Model 1	5.64 (5.02-6.33)^f^
	—	—	—	Model 2	4.17 (3.69-4.72)^f^
	—	—	—	Model 3	1.48 (1.29-1.69)^f^

^a^Absolute prevalence difference was calculated as the prevalence in adolescents with baseline IGD minus the prevalence in adolescents without baseline IGD.

^b^OR: odds ratio.

^c^AOR: adjusted odds ratio.

^d^IGD: internet gaming disorder.

^e^Model 0 was unadjusted.

^f^*P*<.001.

^g^Not applicable.

^h^Model 1 adjusted for demographic variables, including age, sex, age group, only-child status, left-behind status, residence, family type, and parental education.

^i^Model 2 additionally adjusted for lifestyle variables, including alcohol use and tobacco or nicotine use.

^j^Model 3 additionally adjusted for mental health variables, including anxiety, sleep disturbance, depression, hyperactivity and inattention, and conduct problems.

Suicidal ideation was reported by 675 of 1333 adolescents with IGD (50.6%) and 7775 of 56,652 without IGD (13.7%), corresponding to an absolute prevalence difference of 36.9 percentage points (95% CI 34.2-39.6). Suicidal attempt was reported by 527 of 1333 adolescents with IGD (39.5%) and 6139 of 56,652 without IGD (10.8%), corresponding to an absolute prevalence difference of 28.7 percentage points (95% CI 26.1-31.3).

In unadjusted models, IGD was associated with higher odds of suicidal ideation (crude odds ratio [COR] 6.45, 95% CI 5.78-7.20; *P*<.001) and suicidal attempt (COR 5.38, 95% CI 4.81-6.02; *P*<.001). These associations were attenuated after sequential adjustment, particularly after inclusion of mental health covariates. In fully adjusted models, IGD remained significantly associated with suicidal ideation (adjusted odds ratio [AOR] 1.50, 95% CI 1.31-1.72; *P*<.001) and suicidal attempt (AOR 1.48, 95% CI 1.29-1.69; *P*<.001).

### Longitudinal Association Between Baseline IGD and Incident Suicidal Behaviors

As shown in Table 3, among adolescents without suicidal ideation at baseline, 3757 of 49,535 (7.6%) reported incident suicidal ideation at follow-up. Incidence was higher in adolescents with IGD than in those without IGD (16.7%, 110/658 vs 7.5%, 3647/48,877), corresponding to an absolute risk difference of 9.3 percentage points (95% CI 6.4-12.1). IGD was associated with incident suicidal ideation in the unadjusted model (COR 2.49, 95% CI 2.02-3.06; *P*<.001) and remained significant after full adjustment (AOR 1.38, 95% CI 1.11-1.73; *P*=.004).

**Table 3:** Prospective associations between baseline internet gaming disorder and incident suicidal ideation and attempt in a 1-year follow-up cohort of Chinese adolescent gamers in Zigong, China (2021-2022).

Follow-up outcome and group		Outcome, n/N (%)	Absolute risk difference, percentage points (95% CI)	Model	OR^a^ or AOR^b^ (95% CI)
Incident suicidal ideation^c^					
	IGD^d^	110/658 (16.7)	9.3 (6.4-12.1)	Model 0^e^	2.49 (2.02-3.06)^f^
	Non- IGD	3647/48,877 (7.5)	—^g^	Model 1^h^	2.65 (2.15-3.27)^f^
	—	—	—	Model 2^i^	2.33 (1.88-2.88)^f^
	—	—	—	Model 3^j^	1.38 (1.11-1.73)^k^
Incident suicidal attempt^l^					
	IGD	133/806 (16.5)	10.3 (7.7-12.9)	Model 0	2.99 (2.47-3.61)^f^
	Non-IGD	3132/50,513 (6.2)	—	Model 1	3.00 (2.48-3.63)^f^
	—	—	—	Model 2	2.45 (2.02-2.98)^f^
	—	—	—	Model 3	1.37 (1.12-1.68)^k^

^a^OR: odds ratio.

^b^AOR: adjusted odds ratio.

^c^Incident suicidal ideation was defined among adolescents without suicidal ideation at baseline.

^d^IGD: internet gaming disorder.

^e^Model 0 was unadjusted.

^f^*P*<.001.

^g^Not applicable.

^h^Model 1 adjusted for demographic variables, including age, sex, age group, only-child status, left-behind status, residence, family type, and parental education.

^i^Model 2 additionally adjusted for lifestyle variables, including alcohol use and smoking.

^j^Model 3 additionally adjusted for mental health variables, including anxiety, sleep disturbance, depression, hyperactivity and inattention, and conduct problems.

^k^*P*<.01.

^l^Incident suicidal attempt was defined among adolescents without suicidal attempt at baseline.

Among adolescents without a suicidal attempt at baseline, 3265 of 51,319 (6.4%) reported an incident suicidal attempt at follow-up. Incidence was higher in adolescents with IGD than in those without IGD (16.5%, 133/806 vs 6.2%, 3132/50,513), corresponding to an absolute risk difference of 10.3 percentage points (95% CI 7.7-12.9). IGD was associated with incident suicidal attempt in the unadjusted model (COR 2.99, 95% CI 2.47-3.61; *P*<.001) and remained significant after full adjustment (AOR 1.37, 95% CI 1.12-1.68; *P*=.002).

### Prospective Associations of Baseline Suicidal Outcomes With Incident IGD

As shown in Table 4, among adolescents without IGD at baseline, 856 of 56,652 (1.5%) developed IGD at follow-up. Incident IGD was higher among adolescents with baseline suicidal ideation than among those without (3.5%, 271/7775 vs 1.2%, 585/48,877), corresponding to an absolute risk difference of 2.3 percentage points (95% CI 1.9-2.7). Suicidal ideation predicted incident IGD in the unadjusted model (COR 2.98, 95% CI 2.58-3.45; *P*<.001) and remained significant after adjustment (AOR 1.37, 95% CI 1.14-1.64; *P*<.001).

**Table 4:** Prospective associations between baseline suicidal ideation and attempt and incident internet gaming disorder in a 1-year follow-up cohort of Chinese adolescent gamers in Zigong, China (2021-2022).

Baseline exposure and group		Incident IGD^a,b^, n/N (%)	Absolute risk difference^c^, percentage points (95% CI)	Model	OR^d^ or AOR^e^ (95% CI)
Suicidal ideation					
	Exposed	271/7775 (3.5)	2.3 (1.9-2.7)	Model 0^f^	2.98 (2.58-3.45)^g^
	Unexposed	585/48,877 (1.2)	—^h^	Model 1^i^	3.17 (2.72-3.68)^g^
	—	—	—	Model 2^j^	2.75 (2.36-3.22)^g^
	—	—	—	Model 3^k^	1.37 (1.14-1.64)^g^
Suicidal attempt					
	Exposed	204/6139 (3.3)	2.0 (1.6-2.5)	Model 0	2.63 (2.24-3.08)^g^
	Unexposed	652/50,513 (1.3)	—	Model 1	2.94 (2.49-3.46)^g^
	—	—	—	Model 2	2.49 (2.10-2.95)^g^
	—	—	—	Model 3	1.37 (1.14-1.64)^g^

^a^IGD: internet gaming disorder.

^b^Incident probable IGD was defined among adolescents without probable IGD at baseline.

^c^Absolute risk difference was calculated as the incidence in the exposed group minus that in the unexposed group.

^d^OR: odds ratio.

^e^AOR: adjusted odds ratio.

^f^Model 0 was unadjusted.

^g^*P*<.001.

^h^Not applicable.

^i^Model 1 adjusted for demographic variables, including age, sex, age group, only-child status, left-behind status, residence, family type, and parental education.

^j^Model 2 additionally adjusted for lifestyle variables, including alcohol use and tobacco or nicotine use.

^k^Model 3 additionally adjusted for mental health variables, including anxiety, sleep disturbance, depression, hyperactivity and inattention, and conduct problems.

Incident IGD was also higher among adolescents with baseline suicidal attempt than among those without (3.3%, 204/6139 vs 1.3%, 652/50,513), corresponding to an absolute risk difference of 2.0 percentage points (95% CI 1.6-2.5). Suicidal attempt predicted incident IGD in the unadjusted model (COR 2.63, 95% CI 2.24-3.08; *P*<.001) and remained significant after adjustment (AOR 1.37, 95% CI 1.14-1.64; *P*<.001).

### Sensitivity Analysis and Subgroup Analysis

Sensitivity analyses using a lower IGDS9-SF cutoff (≥21) yielded consistent results. Problematic gaming was associated with concurrent suicidal ideation (AOR 1.49, 95% CI 1.40-1.59; *P*<.001) and suicidal attempt (AOR 1.49, 95% CI 1.40-1.59; *P*<.001), and also predicted incident suicidal ideation (AOR 1.38, 95% CI 1.25-1.52; *P*<.001) and suicidal attempt (AOR 1.38, 95% CI 1.24-1.54; *P*<.001). Conversely, baseline suicidal ideation (AOR 1.35, 95% CI 1.23-1.48; *P*<.001) and suicidal attempt (AOR 1.21, 95% CI 1.09-1.34; *P*<.001) were associated with incident problematic gaming.

Subgroup analyses are reported in Supplementary Tables S2-S7. Overall, associations were directionally consistent across sex, age group, only-child status, left-behind status, residence, and family type. Most interaction terms were not statistically significant.

A small number of interaction effects reached nominal significance, including only-child status for suicidal ideation predicting incident IGD and family type for suicidal attempt predicting incident IGD and IGD predicting incident suicidal ideation. Given multiple comparisons, these findings should be interpreted cautiously. Directionality of estimates remained consistent across subgroups, and most nonsignificant subgroup-specific estimates were observed in strata with smaller sample sizes or fewer events.

## Discussion

This large 2-wave cohort study provides longitudinal evidence that IGD and suicidal outcomes are prospectively linked in both directions among Chinese adolescent gamers. Baseline IGD was associated with higher risks of incident suicidal ideation and suicidal attempt over 1 year, while baseline suicidal ideation and suicidal attempt were also associated with subsequent incident IGD. These associations remained evident after sequential adjustment for demographic, lifestyle, and mental health covariates, although the estimates were attenuated after mental health factors were added. By examining suicidal ideation and suicidal attempt separately and testing both temporal directions within the same cohort, this study extends prior work that has largely been cross-sectional or focused only on IGD as a predictor of later suicidality [34-38,53,54]. The findings suggest that IGD and suicidal outcomes may constitute interrelated targets for adolescent mental health screening and prevention.

Our findings were partly consistent with previous literature in a different cultural context [34,38]. In a prospective cohort of Mexican college students, baseline IGD was associated with incident suicidal ideation after adjustment for psychiatric disorders, whereas associations with suicide plans and attempts were not statistically significant [34]. In a Korean adolescent cohort, high risk of IGD predicted subsequent suicidality, suicidal ideation, and suicide plans or attempts among boys, but not among girls [38]. These studies, together with our findings in a large Chinese adolescent sample, suggest that the prospective association between IGD and suicidal outcomes may have relevance across different cultural contexts. However, the pattern of findings has not been uniform. Differences in age range, cultural setting, outcome definition, follow-up design, statistical power, and the number of suicidal attempt events may partly explain the weaker or sex-specific associations reported in previous studies [34,38]. Compared with these earlier cohorts, the present study included a substantially larger sample, providing more precise estimates of the prospective associations with both suicidal outcomes [34,38]. These results also align with a broader literature on the positive association between digital addiction and suicidality [37,55,56], suggesting that addictive engagement with digital platforms may represent an emerging and clinically relevant risk marker for adolescent suicidal risk. From a prevention perspective, these findings support the need to assess problematic digital behaviors, including IGD, alongside conventional mental health risk factors in school-based and clinical screening.

The sequential adjustment models helped clarify the contribution of shared mental health factors to the observed associations. The estimates were substantially attenuated after depression, anxiety, sleep disturbance, hyperactivity and inattention, and conduct problems were added to the models, suggesting that these factors may explain part of the overlap between IGD and suicidal outcomes. Prior studies have linked these psychiatric and behavioral characteristics to IGD, suicidal outcomes, or both, indicating that they may represent shared vulnerabilities or potential pathways connecting the 2 domains [57,58]. Cross-sectional studies have also provided preliminary evidence that mental health and psychosocial factors may mediate the associations between IGD and suicidal outcomes. Yu et al [54] reported that insomnia and depression serially mediated the association between IGD and suicidal ideation, while other studies found that sleep disturbance partially mediated the association between IGD and suicidal behaviors [10,59]. Additional cross-sectional evidence suggests that reduced psychosocial resources and greater psychosocial difficulties, including lower resilience and social support and higher social anxiety and loneliness, may constitute further indirect pathways linking IGD with suicidal ideation [53]. Collectively, these findings support the plausibility of indirect pathways, particularly those involving sleep disturbance and depressive symptoms [10,53,54,59]. However, because the present 2-wave study did not formally test mediation, we could not determine whether these variables functioned as confounders, mediators, or markers of shared vulnerability. The fully adjusted estimates should therefore be interpreted as associations conditional on the measured covariates rather than as total effects. Although the adjusted associations were modest, they remain relevant to clinical and school-based prevention given the severity of suicidal outcomes, the modifiable nature of IGD, and the feasibility of screening for both conditions in adolescent populations.

The most novel contribution of this study is the longitudinal evidence that suicidal outcomes predict the subsequent onset of IGD. Although the precise mechanisms remain unclear, several potential explanations warrant consideration. First, according to the compensatory internet use model [30], adolescents may turn to online gaming as a form of escape-oriented coping when experiencing psychological distress, including suicidal ideation. Repeated reliance on gaming to escape negative emotional states may contribute to excessive and dysregulated gaming, thereby increasing vulnerability to IGD [60-62]. Previous studies have identified escapism as an important gaming motivation associated with both psychological distress and problematic gaming, supporting this interpretation [60-62]. Second, gaming may also serve social motives. Problematic gamers have been reported to endorse stronger social, escape, coping, and fantasy motives than nonproblematic gamers [62]. One possible explanation is that adolescents experiencing suicidal distress may increasingly use gaming environments to seek social connection or validation, thereby increasing their gaming engagement. This interpretation is indirectly supported by a network study by Yang et al [63], in which the item “Do you frequently make new friends online?” showed the strongest association with adolescent suicide risk within the internet addiction symptom network. These mechanisms remain hypotheses because the present study did not assess gaming motives, online social interactions, or the proposed intermediate processes.

This study has several strengths, including its large sample size, prospective design, and adjustment for multiple mental health factors relevant to both IGD and suicidal outcomes. Several limitations should also be noted. First, all measures were based on self-report, which may have introduced recall bias and social desirability bias. Second, the 2-wave, 1-year design limited temporal inference and temporal resolution. Although we examined prospective associations in both directions, 2 waves were insufficient to characterize dynamic reciprocal processes or to separate within-person change from stable between-person differences [64]. The annual interval also lacked the temporal resolution needed to capture short-term fluctuations or acute suicidal crises temporally associated with gaming-related conflict or restriction [16,17]. The findings therefore reflect prospective associations over a 1-year interval rather than dynamic reciprocity or acute effects. In addition, suicidal attempt was assessed as a lifetime item; although incident suicidal attempt was operationalized as no lifetime attempt at baseline and a reported lifetime attempt at follow-up, the exact timing of the attempt during the follow-up interval was not assessed. Third, the sample was drawn from a single Chinese city and was restricted to adolescents who reported gaming in the past 12 months at baseline. The findings therefore apply primarily to adolescent gamers and may not generalize to adolescents without baseline gaming exposure or to populations with different cultural, educational, or mental health service contexts. Fourth, the survey did not collect detailed information on game-related characteristics, such as game genre, violent content, multiplayer format, or social context of play. The findings should therefore be interpreted as associations with overall IGD symptoms rather than effects of specific game types. Finally, school identifiers were removed during data cleaning and were not available for analysis, preventing adjustment for school-level clustering. This may have led to underestimated standard errors and overly narrow CIs. Future studies should use multiwave or intensive longitudinal designs, retain de-identified school-level clustering indicators, and incorporate detailed measures of gaming characteristics, gaming motives, social functioning, and suicide-related mechanisms to clarify when and how IGD and suicidal outcomes become prospectively linked.

In conclusion, this study is innovative in testing prospective associations in both directions within an adolescent gamer cohort while examining suicidal ideation and suicidal attempt separately. Unlike previous studies that were predominantly cross-sectional or treated IGD only as a predictor of later suicidality, it identifies suicidal outcomes as prospective predictors of incident IGD. The findings add longitudinal evidence that IGD and suicidality are interrelated prevention targets, although they do not establish causality. In practice, school-based and clinical screening should assess problematic gaming, suicidal risk, and co-occurring mental health symptoms together, with timely referral and integrated intervention for adolescents who screen positive in either domain.

## Data Availability

The datasets used or analyzed during this study are available from the corresponding author on reasonable request.

## Appendix

Multimedia Appendix 1 Additional analyses of associations between internet gaming disorder and suicidal ideation and suicide attempts.

## Abbreviations

AOR: adjusted odds ratio
COR: crude odds ratio
GAD-7: 7-item Generalized Anxiety Disorder Questionnaire
I-PACE: interaction of person-affect-cognition-execution
IGD: internet gaming disorder
IGDS9-SF: Internet Gaming Disorder Scale-Short Form
MCAR: missing completely at random
PHQ-9: 9-item Patient Health Questionnaire
PSQI: Pittsburgh Sleep Quality Index
SDQ: Strengths and Difficulties Questionnaire

## References

[1] Miller AB, Prinstein MJ (2019). Adolescent suicide as a failure of acute stress-response systems. Annu Rev Clin Psychol.

[2] Zhao M, Li L, Rao Z, Schwebel DC, Ning P, Hu G (2023). Suicide mortality by place, gender, and age group - China, 2010-2021. China CDC Wkly.

[3] Deng Q, Sha L, Hou J, Zhao X, Xiang R, Zhu J, Qu Y, Zhou J, Yu T, Song X, Zheng S, Han T, Yang B, Fan M, Jiang X (2026). Leisure screen time, internet gaming disorder, and mental health among Chinese adolescents: large-scale cross-sectional study. J Med Internet Res.

[4] Li Y, Hu A, Yi L, Mao Z, Lü QY, Wang J, Wei W, Huang Y, Huang S, Dai W, Qiao M, Xu J, Wang Q, Li X, Luo F, Deng W, Hu Y, Li T, Guo W (2026). Comparing the associations of internet addiction and internet gaming disorder with psychopathological symptoms: cross-sectional study of three independent adolescent samples. J Med Internet Res.

[5] Peng P, Chen Z, Ren S, He Y, Li J, Liao A, Zhao L, Shao X, Chen S, He R, Liang Y, Tan Y, Chen X, Tang J, Liao Y (2026). Beyond comorbidity: bidirectional association between sleep problems and internet gaming disorder and the mediating role of resilience and emotional problems: a three-wave cohort study among Chinese adolescents. J Psychiatr Res.

[6] Peng P, Chen Z, Ren S, He Y, Li J, Liao A, Zhao L, Shao X, Chen S, He R, Liang Y, Tan Y, Chen X, Tang J, Liao Y (2026). Peer bullying victimisation and depressive symptoms as serial mediators between attention-deficit/hyperactivity disorder symptoms and internet gaming disorder among Chinese adolescents: a three-wave longitudinal study. Gen Psychiatr.

[7] Peng P, Chen Z, Ren S, He Y, Li J, Liao A, Zhao L, Shao X, Chen S, He R, Liang Y, Tan Y, Chen X, Tang J, Liao Y (2025). Trajectory of internet gaming disorder among Chinese adolescents: course, predictors, and long-term mental health outcomes. J Behav Addict.

[8] Peng P, Jin J, Chen Z, Ren S, He Y, Li J, Liao A, Zhao L, Shao X, Chen S, He R, Liang Y, Tan Y, Chen X, Tang J, Liao Y (2025). Impaired sleep quality mediates the relationship between internet gaming disorder and conduct problems among adolescents: a three-wave longitudinal study. Child Adolesc Psychiatry Ment Health.

[9] Lail SA, Ahmead M (2026). Prevalence of internet gaming disorder, and its association with aggression, impulsivity and suicide ideation among university students. BMC Psychol.

[10] Peng P, Chen Z, Ren S, Liu Y, Li J, Liao A, Zhao L, He R, Liang Y, Tan Y, Tang J, Chen X, Liao Y (2025). Internet gaming disorder and suicidal behaviors mediated by sleep disturbance: a large-scale school-based study in 135,174 Chinese middle school students. Eur Child Adolesc Psychiatry.

[11] Wen L, Xu L, Bai L, Zhu L, Liao L, He R, Liu K, Wang Y (2026). Digital addiction comorbidity patterns and their differential links to internalizing symptoms, suicidal behaviors and non-suicidal self-injury in vocational high school students. BMC Psychol.

[12] Erevik EK, Landrø H, Mattson ÅL, Kristensen JH, Kaur P, Pallesen S (2022). Problem gaming and suicidality: a systematic literature review. Addict Behav Rep.

[13] Junus A, Hsu Y, Wong C, Yip PSF (2023). Is internet gaming disorder associated with suicidal behaviors among the younger generation? Multiple logistic regressions on a large-scale purposive sampling survey. J Psychiatr Res.

[14] Leino T, Finserås TR, Skogen JC, Pallesen S, Kristensen JH, Mentzoni RA, Sivertsen B (2024). Examining the relationship between non-suicidal self-harm and suicidality within the past 12-months and gaming problems in Norwegian full-time students. BMC Psychiatry.

[15] Ohayon MM, Roberts L (2021). Internet gaming disorder and comorbidities among campus-dwelling U.S. university students. Psychiatry Res.

[16] Mamun MA, Griffiths MD (2019). The psychosocial impact of extreme gaming on indian PUBG gamers: The case of PUBG (PlayerUnknown’s Battlegrounds). Int J Ment Health Addiction.

[17] Mamun MA, Ullah I, Usman N, Griffiths MD (2022). PUBG-related suicides during the COVID-19 pandemic: three cases from Pakistan. Perspect Psychiatr Care.

[18] O'Connor RC, Kirtley OJ (2018). The integrated motivational-volitional model of suicidal behaviour. Philos Trans R Soc Lond B Biol Sci.

[19] Kristensen JH, Pallesen S, King DL, Hysing M, Erevik EK (2021). Problematic gaming and sleep: a systematic review and meta-analysis. Front Psychiatry.

[20] Nie Q, Teng Z, Yang C, Griffiths MD, Guo C (2024). Longitudinal relationships between school climate, academic achievement, and gaming disorder symptoms among Chinese adolescents. J Youth Adolesc.

[21] Peng P, Chen ZM, Ren SL, He Y, Li JG, Liao AJ, Zhao LL, Shao X, Chen SS, He RN, Liang YD, Tan YG, Chen XG, Liao YH, Tang JS (2025). Internet gaming disorder and depression mediated by impaired resilience and sleep distress: a three-wave longitudinal study among Chinese adolescents. Epidemiol Psychiatr Sci.

[22] Yu C, Liao X, Ni X, Ou Z, Wang M, Chen J, Huang S (2026). Unraveling the dynamics among internet gaming disorder, depression and emotion dysregulation in Chinese adolescents: a cross-lagged panel network analysis. Addict Behav.

[23] Estupiñá FJ, Bernaldo-de-Quirós M, Vallejo-Achón M, Fernández-Arias I, Labrador F (2024). Emotional regulation in gaming disorder: a systematic review. Am J Addict.

[24] Yu J, Leong Bin Abdullah MFI, Mansor NS (2025). The inhibitory control deficit of internet gaming disorder: an event-related potentials(ERPs) study. Behav Brain Res.

[25] Raybould JN, Tunney RJ (2024). Factor analysis of impulsivity in gaming disorder and internet gaming disorder. BMC Psychiatry.

[26] Nuske J, Nuske L, Stevens MWR, Billieux J, Delfabbro PH, Hides L, Johnson D, King DL (2026). The association between gaming disorder and impulsivity: a systematic review and meta-analysis. Aust N Z J Psychiatry.

[27] Förtsch EM A D, Baumgart P, Teismann T, Ruscheweyh R, Hasenbring MI (2021). No game, more pain - examining possible long term effects and underlying mechanisms of habitual violent video gaming on the acquired capability for suicide. Psychiatry Res.

[28] Mitchell SM, Jahn DR, Guidry ET, Cukrowicz KC (2015). The relationship between video game play and the acquired capability for suicide: an examination of differences by category of video game and gender. Cyberpsychol Behav Soc Netw.

[29] Teismann T, Förtsch E-MAD, Baumgart P, Het S, Michalak J (2014). Influence of violent video gaming on determinants of the acquired capability for suicide. Psychiatry Res.

[30] Kardefelt-Winther D (2014). A conceptual and methodological critique of internet addiction research: towards a model of compensatory internet use. Computers in Human Behavior.

[31] Brand M, Wegmann E, Stark R, Müller A, Wölfling K, Robbins TW, Potenza MN (2019). The interaction of person-affect-cognition-execution (I-PACE) model for addictive behaviors: update, generalization to addictive behaviors beyond internet-use disorders, and specification of the process character of addictive behaviors. Neurosci Biobehav Rev.

[32] Wang P, Pan R, Wu X, Zhu G, Wang Y, Tian M, Sun Y, Wang J (2022). Reciprocal associations between shyness, depression, and internet gaming disorder among Chinese adolescents: a cross-lagged panel study. Addict Behav.

[33] Peng P, Chen Z, Ren S, He Y, Li J, Liao A, Zhao L, Shao X, Chen S, He R, Liang Y, Tan Y, Tang J, Chen X, Liao Y (2026). Sex difference in the longitudinal association between depressive symptoms and internet gaming disorder among Chinese adolescents: an explanatory analysis at the aggregate and symptom level. Addict Behav.

[34] Orozco R, Borges G, Caldas-de-Almeida JM, Gutiérrez-García RA, Albor Y, Jiménez Pérez AL, Valdés-García KP, Baez Mansur PM, Covarrubias Díaz Couder MA, Hernández Uribe PC, Benjet C (2024). Internet gaming disorder and the incidence of suicide-related ideation and behaviors in college students. J Addict Med.

[35] Peng P, Chen Z, Ren S, He Y, Li J, Liao A, Zhao L, Shao X, Chen S, He R, Liang Y, Tan Y, Chen X, Tang J, Liao Y (2025). Internet gaming disorder predicts the incidence, persistence, and worsening of suicidal ideation: a population-based cohort study of 96,158 Chinese adolescents. J Affect Disord.

[36] Peng P, Chen Z, Ren S, Liang Y, Tan Y, Chen X, Tang J, Liao Y (2025). Prospective association of internet gaming disorder with subsequent first suicidal attempt: a large-scale school-based study of Chinese adolescents. Prev Med.

[37] Xiao Y, Meng Y, Brown TT, Keyes KM, Mann JJ (2025). Addictive screen use trajectories and suicidal behaviors, suicidal ideation, and mental health in US youths. JAMA.

[38] Jeong H, Yim HW, Potenza MN, Lee S, Lee HK (2026). Sex differences in the longitudinal association between high risk of internet gaming disorder and subsequent suicidality among adolescents: a cohort study. J Behav Addict.

[39] Chen I, Strong C, Lin Y, Tsai M, Leung H, Lin C, Pakpour AH, Griffiths MD (2020). Time invariance of three ultra-brief internet-related instruments: smartphone application-based addiction scale (SABAS), Bergen Social Media Addiction Scale (BSMAS), and the nine-item internet gaming disorder scale- short form (IGDS-SF9) (Study Part B). Addict Behav.

[40] Pontes HM, Griffiths MD (2015). Measuring DSM-5 internet gaming disorder: development and validation of a short psychometric scale. Computers in Human Behavior.

[41] Qin L, Cheng L, Hu M, Liu Q, Tong J, Hao W, Luo T, Liao Y (2020). Clarification of the cut-off score for nine-item internet gaming disorder scale-short form (IGDS9-SF) in a Chinese context. Front Psychiatry.

[42] Monacis L, Palo VD, Griffiths MD, Sinatra M (2016). Validation of the internet gaming disorder scale - short-form (IGDS9-SF) in an Italian-speaking sample. J Behav Addict.

[43] Yi X, Yi D, Zhang X, Qi W, Zhao M, Li M, Grecucci A, Yuan K, Dong H, Chen BT (2026). Adverse childhood experiences and suicidality in adolescents and young adults with internet gaming disorder: a large-scale school-based study in 13,509 Chinese students. J Affect Disord.

[44] Cheng J, Guan M, Peng C, Hu J, Rong F, Wang Y, Zhang N, Xu Z, Yu Y (2024). Self-injury and suicidal ideation among Chinese adolescents involved in different subtypes of aggression: the role of gender. J Affect Disord.

[45] Lai W, Wu H, Yang L, Chen R, Xin Z, Zhang X, Wang W, Guo L, Huang G, Lu C (2024). Prevalence of unhealthy behaviors and their associations with non-suicidal self-injury, suicidal ideation and suicide attempt among Chinese adolescents. Child Adolesc Psychiatry Ment Health.

[46] Borges G, Nock MK, Haro Abad JM, Hwang I, Sampson NA, Alonso J, Andrade LH, Angermeyer MC, Beautrais A, Bromet E, Bruffaerts R, de Girolamo G, Florescu S, Gureje O, Hu C, Karam EG, Kovess-Masfety V, Lee S, Levinson D, Medina-Mora ME, Ormel J, Posada-Villa J, Sagar R, Tomov T, Uda H, Williams DR, Kessler RC (2010). Twelve-month prevalence of and risk factors for suicide attempts in the World Health Organization world mental health surveys. J Clin Psychiatry.

[47] Zhang YL, Liang W, Chen ZM, Zhang HM, Zhang JH, Weng XQ, Yang SC, Zhang LJ, Shen L, Zhang Y (2013). Validity and reliability of patient health questionnaire-9 and patient health questionnaire-2 to screen for depression among college students in China. Asia Pac Psychiatry.

[48] Sun J, Liang K, Chi X, Chen S (2021). Psychometric properties of the generalized anxiety disorder scale-7 item (GAD-7) in a large sample of Chinese adolescents. Healthcare (Basel).

[49] Tsai PS, Wang SY, Wang MY, Su CT, Yang TT, Huang CJ, Fang SC (2005). Psychometric evaluation of the Chinese version of the Pittsburgh sleep quality index (CPSQI) in primary insomnia and control subjects. Qual Life Res.

[50] Levis B, Benedetti A, Thombs BD, DEPRESsion Screening Data (DEPRESSD) Collaboration (2019). Accuracy of patient health questionnaire-9 (PHQ-9) for screening to detect major depression: individual participant data meta-analysis. BMJ.

[51] Plummer F, Manea L, Trepel D, McMillan D (2016). Screening for anxiety disorders with the GAD-7 and GAD-2: a systematic review and diagnostic metaanalysis. Gen Hosp Psychiatry.

[52] Goodman R (1997). The strengths and difficulties questionnaire: a research note. J Child Psychol Psychiatry.

[53] Yu Y, Wu AMS, Fong VWI, Zhang J, Li J, Lau JTF (2024). Association between internet gaming disorder and suicidal ideation mediated by psychosocial resources and psychosocial problems among adolescent internet gamers in China: cross-sectional study. JMIR Serious Games.

[54] Yu Y, Yang X, Wang S, Wang H, Chang R, Tsamlag L, Zhang S, Xu C, Yu X, Cai Y, Lau JTF (2020). Serial multiple mediation of the association between internet gaming disorder and suicidal ideation by insomnia and depression in adolescents in Shanghai, China. BMC Psychiatry.

[55] Zhao C, He J, Du M, Xu H, Lai X, Yu G, Zhang G (2023). A cross-lagged study of the associations among problematic smartphone use, depressive symptoms, and suicidal ideation in Chinese university students. Curr Psychol.

[56] Li W, Cheng Y, Zhang Z, Yang Y, Jin Y, Cui Z, Wang J, Duan Y, Chen R (2026). Development and validation of a machine learning-based screening tool for early detection of adolescent suicide risk. J Child Psychol Psychiatry.

[57] Richardson R, Connell T, Foster M, Blamires J, Keshoor S, Moir C, Zeng IS (2024). Risk and protective factors of self-harm and suicidality in adolescents: an umbrella review with meta-analysis. J Youth Adolesc.

[58] Zhuang X, Zhang Y, Tang X, Ng TK, Lin J, Yang X (2023). Longitudinal modifiable risk and protective factors of internet gaming disorder: a systematic review and meta-analysis. J Behav Addict.

[59] Garg S, Kharb A, Verma D, Antil R, Khanna B, Sihag R, Lamba D (2023). The mediating role of sleep quality on the relationship between internet gaming disorder and perceived stress and suicidal behaviour among Indian medical students. Gen Psychiatr.

[60] Ballabio M, Griffiths MD, Urbán R, Quartiroli A, Demetrovics Z, Király O (2017). Do gaming motives mediate between psychiatric symptoms and problematic gaming? An empirical survey study. Addiction Research & Theory.

[61] Bányai F, Griffiths MD, Demetrovics Z, Király O (2019). The mediating effect of motivations between psychiatric distress and gaming disorder among esport gamers and recreational gamers. Compr Psychiatry.

[62] Laconi S, Pirès S, Chabrol H (2017). Internet gaming disorder, motives, game genres and psychopathology. Computers in Human Behavior.

[63] Yang Y, Zhang EL, Liu Y, Ge X, Su Z, Cheung T, Ng CH, Xiang M, Xiang YT (2023). Network analysis of suicidality and internet addiction symptoms among Chinese primary and secondary school students. J Affect Disord.

[64] Hamaker EL, Kuiper RM, Grasman RPPP (2015). A critique of the cross-lagged panel model. Psychol Methods.

