# Supplementary material for: Associations in Both Directions Between Internet Gaming Disorder and Suicidal Ideation and Attempts Among Chinese Adolescent Gamers: Two-Wave Prospective Cohort Study
Source: J Med Internet Res. 2026 Sep 2;28:e86919. doi: 10.2196/86919 (PMC13583485; doi:10.2196/86919)
Supplement: Supplementary file 1 [file jmir_v28i1e86919_app1.docx]

Table S1. Group comparison between follow-up and lost-follow-up groups

| **Variables** | **Total (n = 121328)** | **Lost (n = 39945)^1^** | **Follow-up (n = 81383)^1^** | **P^2^** |
| --- | --- | --- | --- | --- |
| **Age, Mean ± SD** | 14.45 ± 1.72 | 15.30 ± 1.89 | 14.03 ± 1.45 | <0.001 |
| **Age Group, n(%)** |  |  |  | **<0.001** |
| Early adolescent | 66376 (54.71) | 14979 (37.50) | 51397 (63.15) |  |
| Late adolescent | 54952 (45.29) | 24966 (62.50) | 29986 (36.85) |  |
| **Sex, n(%)** |  |  |  | <0.001 |
| Boys | 59415 (48.97) | 20284 (50.78) | 39131 (48.08) |  |
| Girls | 61913 (51.03) | 19661 (49.22) | 42252 (51.92) |  |
| **Only child, n(%)** |  |  |  | 0.895 |
| No | 91580 (75.48) | 30142 (75.46) | 61438 (75.49) |  |
| Yes | 29747 (24.52) | 9803 (24.54) | 19944 (24.51) |  |
| **Left-behind child, n(%)** |  |  |  | <0.001 |
| No | 84747 (69.85) | 26882 (67.30) | 57865 (71.10) |  |
| Yes | 36579 (30.15) | 13063 (32.70) | 23516 (28.90) |  |
| **Drinking, n(%)** |  |  |  | <0.001 |
| No | 93852 (77.35) | 29436 (73.69) | 64416 (79.15) |  |
| Yes | 27476 (22.65) | 10509 (26.31) | 16967 (20.85) |  |
| **Smoking, n(%)** |  |  |  | <0.001 |
| No | 110244 (90.86) | 34774 (87.05) | 75470 (92.73) |  |
| Yes | 11084 (9.14) | 5171 (12.95) | 5913 (7.27) |  |
| **Residence, n(%)** |  |  |  | <0.001 |
| Rural | 77093 (63.54) | 27100 (67.84) | 49993 (61.43) |  |
| Urban | 44235 (36.46) | 12845 (32.16) | 31390 (38.57) |  |
| **Family Type , n(%)** |  |  |  | <0.001 |
| Nuclear | 94949 (78.26) | 30720 (76.91) | 64229 (78.92) |  |
| Single-parent | 26379 (21.74) | 9225 (23.09) | 17154 (21.08) |  |
| **Father Education level** |  |  |  | <0.001 |
| Below high school | 92295 (76.07) | 31079 (77.80) | 61216 (75.22) |  |
| High school or above | 29033 (23.93) | 8866 (22.20) | 20167 (24.78) |  |
| **Mother Education level** |  |  |  | <0.001 |
| Below high school | 96054 (79.17) | 32423 (81.17) | 63631 (78.19) |  |
| High school or above | 25274 (20.83) | 7522 (18.83) | 17752 (21.81) |  |
| **Anxiety, n(%)** |  |  |  | <0.001 |
| No | 109418 (90.18) | 35695 (89.36) | 73723 (90.59) |  |
| Yes | 11910 (9.82) | 4250 (10.64) | 7660 (9.41) |  |
| **Sleep Disturbance, n(%)** |  |  |  | <0.001 |
| No | 83965 (69.20) | 26510 (66.37) | 57455 (70.60) |  |
| Yes | 37363 (30.80) | 13435 (33.63) | 23928 (29.40) |  |
| **Depression, n(%)** |  |  |  | <0.001 |
| No | 100600 (82.92) | 32523 (81.42) | 68077 (83.65) |  |
| Yes | 20728 (17.08) | 7422 (18.58) | 13306 (16.35) |  |
| **ADHD, n(%)** |  |  |  | <0.001 |
| No | 109751 (90.46) | 35784 (89.58) | 73967 (90.89) |  |
| Yes | 11577 (9.54) | 4161 (10.42) | 7416 (9.11) |  |
| **Conduct problems, n(%)** |  |  |  | <0.001 |
| No | 112265 (92.53) | 36672 (91.81) | 75593 (92.89) |  |
| Yes | 9063 (7.47) | 3273 (8.19) | 5790 (7.11) |  |
| **IGD, n(%)** |  |  |  | <0.001 |
| No | 105126 (86.65) | 34047 (85.23) | 71079 (87.34) |  |
| Yes | 16202 (13.35) | 5898 (14.77) | 10304 (12.66) |  |
| **Suicidal behaviors, n(%)** |  |  |  | <0.001 |
| No | 99258 (81.81) | 32290 (80.84) | 66968 (82.29) |  |
| Yes | 22070 (18.19) | 7655 (19.16) | 14415 (17.71) |  |

1Mean (SD); n (%)

2t test; Pearson's Chi-squared test

Supplementary Table S2. Subgroup analyses for the cross-sectional association between IGD and suicidal ideation

| Variables | n (%) | Non-IGD | IGD | AOR (95%CI) | *P* | P for interaction |
| --- | --- | --- | --- | --- | --- | --- |
|  |  |  |  |  |  |  |
| Sex |  |  |  |  |  | 0.120 |
| Boys | 31643 (54.57) | 2749/30875 | 301/768 | 1.40 (1.16 ~ 1.67) | **<0.001** |  |
| Girls | 26342 (45.43) | 5026/25777 | 374/565 | 1.67 (1.36 ~ 2.06) | **<0.001** |  |
| Only Child |  |  |  |  |  | 0.610 |
| No | 43264 (74.61) | 5797/42310 | 479/954 | 1.48 (1.26 ~ 1.74) | **<0.001** |  |
| Yes | 14721 (25.39) | 1978/14342 | 196/379 | 1.57 (1.22 ~ 2.03) | **<0.001** |  |
| Left behind |  |  |  |  |  | 0.411 |
| No | 40922 (70.57) | 5467/40051 | 432/871 | 1.45 (1.23 ~ 1.72) | **<0.001** |  |
| Yes | 17063 (29.43) | 2308/16601 | 243/462 | 1.59 (1.26 ~ 2.00) | **<0.001** |  |
| Residence |  |  |  |  |  | 0.108 |
| Rural | 35300 (60.88) | 4547/34502 | 407/798 | 1.63 (1.36 ~ 1.94) | **<0.001** |  |
| Urban | 22685 (39.12) | 3228/22150 | 268/535 | 1.34 (1.08 ~ 1.65) | **0.008** |  |
| Nuclear Family |  |  |  |  |  | 0.589 |
| No | 45063 (77.71) | 5648/44164 | 435/899 | 1.50 (1.27 ~ 1.77) | **<0.001** |  |
| Yes | 12922 (22.29) | 2127/12488 | 240/434 | 1.50 (1.19 ~ 1.91) | **<0.001** |  |
| Age group |  |  |  |  |  | 0.314 |
| Early adolescent | 35182 (60.67) | 5284/34286 | 502/896 | 1.51 (1.27 ~ 1.79) | **<0.001** |  |
| Late adolescent | 22803 (39.33) | 2491/22366 | 173/437 | 1.44 (1.15 ~ 1.80) | **0.002** |  |
| OR: Odds Ratio, CI: Confidence Interval  Adjusted for baseline demographics and mental health issues (depression, anxiety, sleep distress, and externalizing problems) | | | | | | |

Supplementary Table S3. Subgroup analyses for the cross-sectional association between IGD and suicidal attempt

| Variables | n (%) | Non-IGD | IGD | AOR (95%CI) | *P* | P for interaction |
| --- | --- | --- | --- | --- | --- | --- |
|  |  |  |  |  |  |  |
| Sex |  |  |  |  |  | 0.807 |
| Boys | 31643 (54.57) | 2237/30875 | 239/768 | 1.49 (1.23 ~ 1.79) | **<0.001** |  |
| Girls | 26342 (45.43) | 3902/25777 | 288/565 | 1.49 (1.23 ~ 1.80) | **<0.001** |  |
| Only Child |  |  |  |  |  | 0.160 |
| No | 43264 (74.61) | 4677/42310 | 392/954 | 1.58 (1.35 ~ 1.85) | **<0.001** |  |
| Yes | 14721 (25.39) | 1462/14342 | 135/379 | 1.26 (0.98 ~ 1.62) | 0.076 |  |
| Left behind |  |  |  |  |  | 0.652 |
| No | 40922 (70.57) | 4242/40051 | 341/871 | 1.51 (1.28 ~ 1.78) | **<0.001** |  |
| Yes | 17063 (29.43) | 1897/16601 | 186/462 | 1.43 (1.14 ~ 1.79) | **0.002** |  |
| Residence |  |  |  |  |  | 0.855 |
| Rural | 35300 (60.88) | 3767/34502 | 319/798 | 1.50 (1.27 ~ 1.78) | **<0.001** |  |
| Urban | 22685 (39.12) | 2372/22150 | 208/535 | 1.45 (1.18 ~ 1.79) | **<0.001** |  |
| Nuclear Family |  |  |  |  |  | 0.447 |
| No | 45063 (77.71) | 4425/44164 | 339/899 | 1.50 (1.27 ~ 1.76) | **<0.001** |  |
| Yes | 12922 (22.29) | 1714/12488 | 188/434 | 1.47 (1.17 ~ 1.84) | **0.001** |  |
| Age group |  |  |  |  |  | 0.306 |
| Early adolescent | 35182 (60.67) | 3710/34286 | 378/896 | 1.44 (1.22 ~ 1.70) | **<0.001** |  |
| Late adolescent | 22803 (39.33) | 2429/22366 | 149/437 | 1.49 (1.19 ~ 1.86) | **<0.001** |  |
| OR: Odds Ratio, CI: Confidence Interval  Adjusted for baseline demographics and mental health issues (depression, anxiety, sleep distress, and externalizing problems) | | | | | | |

Supplementary Table S4. Subgroup analyses for the predictive effect of SI on incident IGD

| Variables | n (%) | Non-SI | SI | OR (95%CI) | *P* | P for interaction |
| --- | --- | --- | --- | --- | --- | --- |
|  |  |  |  |  |  |  |
| Sex |  |  |  |  |  | 0.455 |
| Boys | 30875 (54.50) | 418/28126 | 134/2749 | 1.35 (1.07 ~ 1.70) | **0.012** |  |
| Girls | 25777 (45.50) | 167/20751 | 137/5026 | 1.43 (1.08 ~ 1.89) | **0.013** |  |
| Only Child |  |  |  |  |  | 0.030 |
| No | 42310 (74.68) | 381/36513 | 195/5797 | 1.49 (1.20 ~ 1.85) | **<0.001** |  |
| Yes | 14342 (25.32) | 204/12364 | 76/1978 | 1.14 (0.82 ~ 1.58) | 0.427 |  |
| Left behind |  |  |  |  |  | 0.478 |
| No | 40051 (70.70) | 435/34584 | 191/5467 | 1.27 (1.03 ~ 1.56) | **0.027** |  |
| Yes | 16601 (29.30) | 150/14293 | 80/2308 | 1.68 (1.20 ~ 2.37) | **0.003** |  |
| Residence |  |  |  |  |  | 0.095 |
| Rural | 34502 (60.90) | 314/29955 | 155/4547 | 1.47 (1.16 ~ 1.87) | **0.002** |  |
| Urban | 22150 (39.10) | 271/18922 | 116/3228 | 1.24 (0.95 ~ 1.62) | 0.119 |  |
| Nuclear Family |  |  |  |  |  | 0.115 |
| No | 44164 (77.96) | 413/38516 | 185/5648 | 1.35 (1.09 ~ 1.68) | **0.006** |  |
| Yes | 12488 (22.04) | 172/10361 | 86/2127 | 1.42 (1.03 ~ 1.96) | **0.032** |  |
| Age group |  |  |  |  |  | 0.098 |
| Early adolescent | 34286 (60.52) | 410/29002 | 224/5284 | 1.61 (1.31 ~ 1.99) | **<0.001** |  |
| Late adolescent | 22366 (39.48) | 175/19875 | 47/2491 | 0.88 (0.61 ~ 1.27) | 0.511 |  |

OR: Odds Ratio, CI: Confidence Interval

Adjusted for baseline demographics and mental health issues (depression, anxiety, sleep distress, and externalizing problems)

Supplementary Table S5. Subgroup analyses for the predictive effect of SI on incident IGD

| Variables | n (%) | Non-SA | SA | AOR (95%CI) | *P* | P for interaction |  |
| --- | --- | --- | --- | --- | --- | --- | --- |
|  |  |  |  |  |  |  |  |
| Sex |  |  |  |  |  | 0.722 |  |
| Boys | 30875 (54.50) | 459/28638 | 93/2237 | 1.25 (0.97 ~ 1.60) | 0.082 |  |  |
| Girls | 25777 (45.50) | 193/21875 | 111/3902 | 1.56 (1.19 ~ 2.05) | **0.001** |  |  |
| Only Child |  |  |  |  |  | 0.214 |  |
| No | 42310 (74.68) | 430/37633 | 146/4677 | 1.41 (1.14 ~ 1.76) | **0.002** |  |  |
| Yes | 14342 (25.32) | 222/12880 | 58/1462 | 1.27 (0.91 ~ 1.78) | 0.156 |  |  |
| Left behind |  |  |  |  |  | 0.893 |  |
| No | 40051 (70.70) | 479/35809 | 147/4242 | 1.35 (1.09 ~ 1.67) | **0.006** |  |  |
| Yes | 16601 (29.30) | 173/14704 | 57/1897 | 1.44 (1.02 ~ 2.04) | **0.038** |  |  |
| Residence |  |  |  |  |  | 0.053 |  |
| Rural | 34502 (60.90) | 345/30735 | 124/3767 | 1.55 (1.22 ~ 1.97) | **<0.001** |  |  |
| Urban | 22150 (39.10) | 307/19778 | 80/2372 | 1.17 (0.88 ~ 1.55) | 0.286 |  |  |
| Nuclear Family |  |  |  |  |  | 0.006 |  |
| No | 44164 (77.96) | 451/39739 | 147/4425 | 1.50 (1.21 ~ 1.87) | **<0.001** |  |  |
| Yes | 12488 (22.04) | 201/10774 | 57/1714 | 1.10 (0.78 ~ 1.55) | 0.582 |  |  |
| Age group |  |  |  |  |  | 0.102 |  |
| Early adolescent | 34286 (60.52) | 489/30576 | 145/3710 | 1.28 (1.03 ~ 1.59) | **0.025** |  |  |
| Late adolescent | 22366 (39.48) | 163/19937 | 59/2429 | 1.60 (1.15 ~ 2.24) | **0.006** |  |  |
| OR: Odds Ratio, CI: Confidence Interval | | | | | | |  |

Adjusted for baseline demographics and mental health issues (depression, anxiety, sleep distress, and externalizing problems)

Supplementary Table S6. Subgroup analyses for the predictive effect of IGD on incident SI

| Variables | n (%) | Non-IGD | IGD | AOR (95%CI) | *P* | P for interaction |
| --- | --- | --- | --- | --- | --- | --- |
|  |  |  |  |  |  |  |
| Sex |  |  |  |  |  | 0.482 |
| Boys | 28593 (57.72) | 1652/28126 | 71/467 | 1.42 (1.08 ~ 1.88) | **0.013** |  |
| Girls | 20942 (42.28) | 1995/20751 | 39/191 | 1.26 (0.87 ~ 1.83) | 0.224 |  |
| Only Child |  |  |  |  |  | 0.824 |
| No | 36988 (74.67) | 2644/36513 | 76/475 | 1.36 (1.04 ~ 1.77) | **0.023** |  |
| Yes | 12547 (25.33) | 1003/12364 | 34/183 | 1.45 (0.97 ~ 2.17) | 0.073 |  |
| Left behind |  |  |  |  |  | 0.134 |
| No | 35023 (70.70) | 2622/34584 | 69/439 | 1.25 (0.95 ~ 1.65) | 0.111 |  |
| Yes | 14512 (29.30) | 1025/14293 | 41/219 | 1.67 (1.15 ~ 2.42) | **0.006** |  |
| Residence |  |  |  |  |  | 0.780 |
| Rural | 30346 (61.26) | 2148/29955 | 60/391 | 1.28 (0.95 ~ 1.73) | 0.098 |  |
| Urban | 19189 (38.74) | 1499/18922 | 50/267 | 1.54 (1.10 ~ 2.15) | **0.011** |  |
| Nuclear Family |  |  |  |  |  | 0.030 |
| No | 38980 (78.69) | 2740/38516 | 82/464 | 1.60 (1.24 ~ 2.08) | **<0.001** |  |
| Yes | 10555 (21.31) | 907/10361 | 28/194 | 0.97 (0.63 ~ 1.50) | 0.905 |  |
| Age group |  |  |  |  |  | 0.361 |
| Early adolescent | 29396 (59.34) | 2451/29002 | 78/394 | 1.57 (1.20 ~ 2.06) | **0.001** |  |
| Late adolescent | 20139 (40.66) | 1196/19875 | 32/264 | 1.09 (0.74 ~ 1.62) | 0.663 |  |
| OR: Odds Ratio, CI: Confidence Interval | | | | | | |

Adjusted for baseline demographics and mental health issues (depression, anxiety, sleep distress, and externalizing problems)

Supplementary Table S6. Subgroup analyses for the predictive effect of IGD on incident SA

| Variables | n (%) | Non-IGD | IGD | OR (95%CI) | *P* | P for interaction |
| --- | --- | --- | --- | --- | --- | --- |
|  |  |  |  |  |  |  |
| Sex |  |  |  |  |  | 0.122 |
| Boys | 29167 (56.83) | 1525/28638 | 84/529 | 1.58 (1.22 ~ 2.05) | **<0.001** |  |
| Girls | 22152 (43.17) | 1607/21875 | 49/277 | 1.09 (0.78 ~ 1.53) | 0.597 |  |
| Only Child |  |  |  |  |  | 0.482 |
| No | 38195 (74.43) | 2330/37633 | 88/562 | 1.30 (1.02 ~ 1.67) | **0.036** |  |
| Yes | 13124 (25.57) | 802/12880 | 45/244 | 1.55 (1.09 ~ 2.22) | **0.016** |  |
| Left behind |  |  |  |  |  | 0.348 |
| No | 36339 (70.81) | 2153/35809 | 80/530 | 1.24 (0.95 ~ 1.60) | 0.109 |  |
| Yes | 14980 (29.19) | 979/14704 | 53/276 | 1.62 (1.17 ~ 2.26) | **0.004** |  |
| Residence |  |  |  |  |  | 0.469 |
| Rural | 31214 (60.82) | 1925/30735 | 83/479 | 1.44 (1.11 ~ 1.87) | **0.006** |  |
| Urban | 20105 (39.18) | 1207/19778 | 50/327 | 1.27 (0.91 ~ 1.76) | 0.159 |  |
| Nuclear Family |  |  |  |  |  | 0.134 |
| No | 40299 (78.53) | 2332/39739 | 93/560 | 1.48 (1.16 ~ 1.89) | **0.002** |  |
| Yes | 11020 (21.47) | 800/10774 | 40/246 | 1.21 (0.84 ~ 1.75) | 0.313 |  |
| Age group |  |  |  |  |  | 0.455 |
| Early adolescent | 31094 (60.59) | 1996/30576 | 86/518 | 1.32 (1.03 ~ 1.71) | **0.031** |  |
| Late adolescent | 20225 (39.41) | 1136/19937 | 47/288 | 1.46 (1.04 ~ 2.05) | **0.028** |  |
| OR: Odds Ratio, CI: Confidence Interval | | | | | | |

Adjusted for baseline demographics and mental health issues (depression, anxiety, sleep distress, and externalizing problems)
